# Book on the Physician Himself, and Things That Concern His Reputation and Success

**Published:** 1903-09

**Authors:** 


Book on the Physician Himself, and Things that Concern His Reputation and Success.—By D. W. Cathell, M. D. The Twentieth Century edition, being the eleventh edition, revised and enlarged by the author and his son, William T. Cathell, A. M., M. D. Pages, 411, royal octavo, extra cloth, $2.50 net, delivered. Philadelphia: F. A. Davis, Publishers, 1914-16 Cherry Street.

A careful reading of this book will benefit the old, as well as the young, physician. It is the code of medical ethics amplified, and is based on the golden rule. The young physician should know early in his professional career how to view humanity broadly, how to take a steady course for right and justice, and how to practice with his heart in one hand and his ledger in the other. This book teaches him how to do it. The older physician, though a failure, has still time to learn how to succeed in his latter days. This book teaches him how it is done.

It has been revised up to date, and will benefit all who read it.

